# *Dioscorea esculenta* Intake with Resistance Training Improves Muscle Quantity and Quality in Healthy Middle-Aged and Older Adults: A Randomized Controlled Trial

**DOI:** 10.3390/nu15112438

**Published:** 2023-05-24

**Authors:** Keiko Iemitsu, Shumpei Fujie, Masataka Uchida, Kenichiro Inoue, Yasushi Shinohara, Motoyuki Iemitsu

**Affiliations:** 1 Faculty of Sport and Health Science, Ritsumeikan University, Kusatsu 525-8577, Japan; kiemitsu@fc.ritsumei.ac.jp (K.I.); s-fujie@fc.ritsumei.ac.jp (S.F.); m-uchida@fc.ritsumei.ac.jp (M.U.); sh0118es@ed.ritsumei.ac.jp (K.I.); ysr15159@fc.ritsumei.ac.jp (Y.S.); 2 Japan Society for the Promotion of Science, Tokyo 102-0083, Japan

**Keywords:** *Dioscorea esculenta*, resistance exercise, muscle thickness, muscle echo intensity, muscle function, older adults

## Abstract

Resistance training and *Dioscorea esculenta* intake have a positive effect on muscle. Therefore, we aimed to determine whether 12-week *Dioscorea esculenta* intake combined with resistance exercise more effectively improves muscle quantity, quality, and cardiometabolic parameters in healthy middle-aged and older adults. This study is a double-blind trial with 66 volunteers (21 males/45 females; age 53 ± 5 years; body weight 61 ± 11 kg; BMI 24 ± 4 kg) who were randomly divided into four groups: sedentary-control with placebo (Sed and PL) or Dioscorea (Sed and Dio) and resistance training with placebo (RT and PL) or Dioscorea (RT and Dio). Resistance training sessions using elastic bands were performed 3 days/week for a 12-week period. *Dioscorea esculenta* tablets were ingested at 2000 mg/day once per day. The RT and Dio group showed greater improvements in the femoris muscle’s thickness, echo intensity for the rectus femoris (index of muscle quality), and the five times sit-to-stand test compared to that of the Sed and PL group; the echo intensity in the RT and Dio group further improved compared to those in the Sed and Dio, and RT and PL groups (*p* < 0.05). The circulating levels of C1q (a potential biomarker of muscle fibrosis) in the RT and Dio group were significantly lower than those in the Sed and PL, and Sed and Dio groups (*p* < 0.05). Chronic *Dioscorea esculenta* intake combined with low-intensity resistance exercise may more effectively improve muscle quantity and quality indices in healthy middle-aged and older adults.

## 1. Introduction

Aging reduces muscle mass and strength and increases all-cause morbidity and mortality [1,2,3]. Age-related decline in muscle mass and strength accelerates the development of chronic diseases, such as hypertension, diabetes, osteoporosis, and hyperlipidemia, which are the leading causes of physical disability and frailty [3,4,5,6]. Moreover, aging increases muscle adipose and fibrous connective tissues with a loss of muscle mass, leading to lower muscle quality [7,8]. However, habitual resistance exercise can improve muscle quantity and quality, increasing muscle mass and function and reducing muscle fibrosis [9,10].

The secretion of sex steroid hormones by the testes, ovaries, and adrenal cortex and the provision of sex steroid hormones to various peripheral tissues are traditionally well known. Additionally, androgen hormones that contain dehydroepiandrosterone (DHEA), testosterone, and dihydrotestosterone (DHT) regulate anabolism [11], insulin sensitivity [12,13], muscle energy metabolism [14,15], bone turnover [16], and anti-stress effects [17]. Our previous studies using in vitro and in vivo showed that DHEA, which is a steroid hormone precursor, was metabolized to testosterone using steroidogenic enzymes, such as 3β-hydroxysteroid dehydrogenase (HSD) and 17β-HSD, and testosterone was converted to DHT via 5α-reductase in skeletal muscle, indicating that skeletal muscle can synthesize sex steroid hormones [15,18]. In our recent reports, androgen hormones in the skeletal muscle were found to play a novel role in upregulating muscle glucose, lipid metabolism, and muscle protein synthesis [14,15]. However, the sex steroid hormone secretion capacity is impaired with age [19,20]. Habitual resistance exercise increases muscle mass, androgen hormone, and steroidogenic enzyme expression levels in elderly adults, and increased muscle DHT secretion is correlated with muscle hypertrophy [20]. Furthermore, our previous study using rodent models showed that resistance training induced muscle hypertrophy and improved glycemic control; however, treatment with a DHT inhibitor suppressed these effects [21]. Therefore, the resistance training-induced secretion of androgen hormones is an important compound related to muscle mass and cardiometabolic parameters. 

*Dioscorea esculenta* is commonly known as lesser yam. The tubers of *Dioscorea esculenta* contain high levels of a natural product: diosgenin. Diosgenin, which is a plant-derived steroidal saponin produced via saponin hydrolysis, contains a molecular formula similar to DHEA [22]. Our previous study using rodent models demonstrated that acute or chronic administration of *Dioscorea esculenta* improved glycemic control via activation of the muscle GLUT-4 signaling pathway with increased muscle sex steroid hormone levels in diabetic rats [23]. Furthermore, in a recent study, chronic *Dioscorea esculenta* tablet intake at 2000 mg/day combined with resistance exercise further increased muscle mass and strength with increased androgen hormone secretion in athletes [24]. However, the effects of chronic *Dioscorea esculenta* consumption combined with resistance exercise on muscle quantity, muscle quality, and cardiometabolic parameters in middle-aged and older adults remain unclear.

This study aimed to clarify whether *Dioscorea esculenta* supplementation combined with resistance exercise more effectively improves muscle quantity, muscle quality, and cardiometabolic parameters, especially the glycemic control index, in healthy middle-aged and older adults. To address this hypothesis, we used a randomized controlled intervention trial to examine the effects of 12 weeks of *Dioscorea esculenta* tablet intake combined with low-intensity resistance exercise on muscle thickness, muscle function, muscle quality index (intramuscular fibrosis and fat infiltration), and cardiometabolic parameters such as blood pressure, glycated hemoglobin A1c (HbA1c), and lipid profiles.

## 2. Materials and Methods

### 2.1. Participants

We recruited middle-aged and elderly community-dwelling adults from Japan. Ninety-eight participants were recruited through advertisements published in the local press and posters at community health and recreation centers. Thirty-two individuals who were performing habitual exercise; were taking anti-hyperlipidemic, anti-hypertensive, or anti-hyperglycemic medication; or had a history of stroke, diabetes, hypertension, hyperlipidemia, cardiac disease, chronic renal failure, gynecological disease, joint disorder, or mental disorder were excluded from this study. Sixty-six healthy middle-aged and older sedentary participants volunteered to participate in this study. 

All the participants voluntarily provided written informed consent before participating in the study. This study was approved by the Ethics Committee of Ritsumeikan University and conducted in accordance with the Declaration of Helsinki. The study was registered in the University Hospital Medical Information Network Clinical Trials Registry (UMIN-CTR; UMIN000045605).

### 2.2. Study Design

After informed consent for participation in the study was obtained, allocation was performed using computer-generated random numbers for each sex. In this study, using a randomized controlled intervention trial with a double-blind study design, participants were randomly divided into four groups: the sedentary-control and placebo intake (Sed and PL) group, the sedentary-control and *Dioscorea* intake (Sed and Dio) group, the resistance training and placebo intake (RT and PL) group, and the resistance training and *Dioscorea* intake (RT and Dio) group. The assessors who measured these parameters were blinded to the group allocation in this study. Body composition, muscle quantity and quality indices, cardiometabolic parameters, physical activity, and energy intake were measured before and after the 12-week resistance training program. Before and after each intervention period, blood samples were collected in the morning (8:00–9:30 a.m.) at least 48 h after the last resistance training session, avoiding immediate acute effects of exercise. All participants were instructed not to eat or drink fluids other than water for at least 12 h before blood sampling. The experimental room temperature was maintained at 24 °C both before and after intervention measurements. All participants were instructed to maintain their physical activity and food intake during the duration of the experiment, excluding *Dioscorea* and/or placebo intake and/or resistance training.

### 2.3. Resistance Training Intervention

Participants performed resistance training sessions using elastic bands (THERABAND, The Hygenic Corporation, Akron, OH, USA) in each participant’s home in the evening (7:00–8:00 p.m.); sessions took place 3 days per week on non-consecutive days for 12 weeks. Participants were supervised by experienced trainers in all training sessions with a remote control using Zoom (Zoom Video Communications, San Jose, CA, USA) to ensure proper intensity, exercise technique, rest intervals, and progression during each exercise session. Each session lasted 60 min and constructed of 5-min warm-ups, 50-min resistance elastic band exercises, and 5-min cool-downs. The resistance exercise protocol using an elastic band was designed for 10 exercises for the trunk and limb muscle groups, including the arms, shoulder, abdomen, back, and legs, according to the guidelines of the American College of Sports Medicine [25,26]. Participants performed 1–2 sets of 8–12 repetitions during the first 3 weeks and 3 sets of 8–12 repetitions during the rest of the study. Exercise intensity was maintained on a scale of 12–14 points for each participant using the Borg scale, which gradually increased during exercise training; therefore, the resistance levels of the elastic band gradually increased from the lowest yellow to red (or blue) color. In this study, the mean Borg scale during the resistance exercise training intervention period was 13 ± 1. In this study, if the rate of participation in 36 resistance training sessions was 90% or less, the individual was excluded from the statistical analysis due to their “Low adherence to the exercise”.

### 2.4. Dioscorea esculenta or Placebo Intake 

*Dioscorea esculenta* or placebo tablets were administered in a double-blind placebo-controlled study [24]. *Dioscorea esculenta* tablets (OTV Development Co., Ltd., Okinawa, Japan) were ingested at 2000 mg/day once per day within 30 min of eating dinner. The placebo tablet contained cornstarch with the same caloric intake as the *Dioscorea esculenta* tablet [27] and was ingested at 2000 mg/day, once a day, within 30 min of eating dinner. In this study, if the intake rate of *Dioscorea esculenta* or placebo tablets during intervention was less than 95%, the individual was excluded from the statistical analysis due to their “Low adherence to the nutrition”.

### 2.5. Outcome Measures

#### 2.5.1. Body Composition

Body weight and height were measured to the nearest 0.1 cm and 0.1 kg, respectively. Body mass index (BMI; kg/m^2^) was calculated using these two variables. Body weight and percent body fat were measured using a body composition analyzer (RD-801; TANITA, Tokyo, Japan).

#### 2.5.2. Physical Activity, Energy Intake, and Nutritional Status

The physical activity levels were assessed using the short form of the International Physical Activity Questionnaire (IPAQ-SF), as previously described [28]. Before and after each intervention, dietary energy intake and nutritional status was assessed using a validated brief self-administered diet history questionnaire composed of 73 items based on a self-administered diet history questionnaire, as previously described [29].

#### 2.5.3. Muscle Quantity and Quality Indexes 

To assess the muscle thicknesses of the anterior and posterior femoris muscles and the echo intensity of the rectus femoris muscle, we used a B-mode ultrasound device with 3.4–8.0 MHz linear-array probe (Vscan dual probe, GE Healthcare, Chicago, IL, USA). Ultrasound images consistently set the following parameters: frequency 8.0 MHz; gain 60 dB; depth 6–10 cm. All participants were scanned perpendicularly on the anterior and posterior thigh surfaces, halfway between the lateral condyle of the femur and the greater trochanter, in the anatomical standing position, with their arms and legs extended and relaxed. Three images of the anterior and posterior femoris muscles were obtained for each measurement site and stored on a personal computer. Images obtained from an ultrasound device regarding muscle thickness and echo intensity were analyzed using ImageJ (version 1.52a; National Institutes of Health, Bethesda, MD, USA), as previously reported [30,31]. The thicknesses of the anterior and posterior femoris muscles were measured as the distances between the inferior edge of the superficial aponeurosis, which is located between the subcutaneous fat and skeletal muscle, and the superior edge of the femur. Echo intensity can be used to objectively evaluate intramuscular fibrosis and fat infiltration. For the rectus femoris muscle echo intensity, the region of interest (ROI) was set for the rectus femoris while avoiding the fascia. The echo intensity of the ROI was showed in arbitrary units as values between 0 and 255 (black = 0 and 255 = white = 255), and these values were adjusted according to the area of the rectus femoris ROI. For each analyzed muscle, representative muscle thickness and echo intensity were taken as the mean of three measurements.

As indices of muscle function, 6-m normal walking speed, single-leg stand test, five times sit-to-stand test, and handgrip strength were assessed before and after each intervention, as previously reported [32]. All measurements were performed by a trained trainer. For each test, the representative value of these parameters was taken as the average of two measurements.

#### 2.5.4. Cardiometabolic Parameters

All participants sat quietly for 30 min, and brachial systolic (SBP) and diastolic (DBP) blood pressures and heart rate (HR) were measured in duplicate in the sitting position at rest (HEM-7120, OMRON Healthcare, Kyoto, Japan).

The sample in serum was immediately collected via centrifuge (1500× *g*, 4 °C, 15 min). Blood samples were stored at −80 °C until use. The room temperature was maintained at 22–24 °C during the analysis. Fasting levels of total cholesterol (Total-Cho), high-density lipoprotein cholesterol (HDL), and triglyceride (TG) were measured using standard enzymatic techniques, as previously described [33]. The levels of HbA1c were measured using an enzymatic assay. 

### 2.6. Statistical Analysis

All values were expressed as mean ± standard deviation (SD). Statistical analysis of each parameter was carried out using two-way analysis of variance (ANOVA) for repeated measures (time × group). One-way ANOVA was used to compare the differences in changes from baseline to 12 weeks among the four groups. A post hoc comparison test was used to correct for multiple comparisons (two-tailed Fisher’s post hoc test) when the analyses revealed significant differences. The relationships between changes in serum C1q levels and echo intensity in the rectus femoris from baseline to 12-week interventions were determined via Pearson’s correlation coefficients. Statistical significance was defined as (*p* < 0.05). All statistical analyses were performed using the StatView (ver. 5.0; SAS Institute, Minato-ku, Tokyo, Japan).

The required sample size for using repeated-measures ANOVA to examine comparisons between the four groups before and after the 12-week intervention with α set at 0.05 and power at 0.80 was 48 participants (12 in each group), as calculated using G*Power (ver. 3.1.9.6).

## 3. Results

### 3.1. Participant Characteristics and Nutritional Status

**Figure 1:**
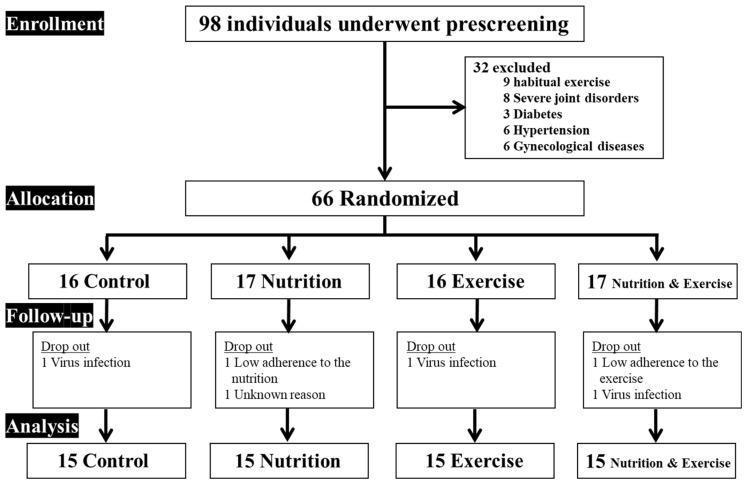
Participant flow chart.

The participant flowchart for this study is shown in Figure 1. Overall, 98 middle-aged and older adults were screened and 66 participants were enrolled in the study (total: 53 ± 5 years; males: *n* = 21, 54 ± 6 years; females: *n* = 45, 53 ± 5 years; Sed and PL group: *n* = 16 [5 males, 11 females], Sed and Dio group: *n* = 17 [5 males, 12 females], RT and PL group: *n* = 16 [5 males, 11 females], and RT and Dio group: *n* = 17 [6 males, 11 females]). A total of 6 participants dropped out at follow-up, while 60 individuals completed the outcome measurements before and after the interventions (each *n* = 15, Sed and PL group [88.2%, 4 males, 11 females], Sed and Dio group [93.8%, 4 males, 11 females], RT and PL group [88.2%, 4 males, 11 females], and RT and Dio group [93.8%, 4 males, 11 females]) (Figure 1). No direct adverse events resulting from this intervention were reported. The average attendance rate for the 36 resistance training sessions in this study was 94%. The average intake rate of *Dioscorea esculenta* or placebo during the intervention was 98%.

No significant differences in age, height, body weight, BMI, percent body fat, daily physical activity, or daily total energy intake were observed among the four groups before and after the intervention (Table 1). Furthermore, changes in body weight, BMI, body fat, daily physical activity, and daily total energy intake from baseline to 12 weeks did not differ among the four groups (Table 1).

Additionally, no significant differences in the main nutritional statuses were observed among the four groups before and after the intervention (Table 2). Furthermore, changes in nutritional status from baseline to 12 weeks did not differ among the four groups (Table 2).

### 3.2. Muscle Quantity and Quality Indices

**Figure 2:**
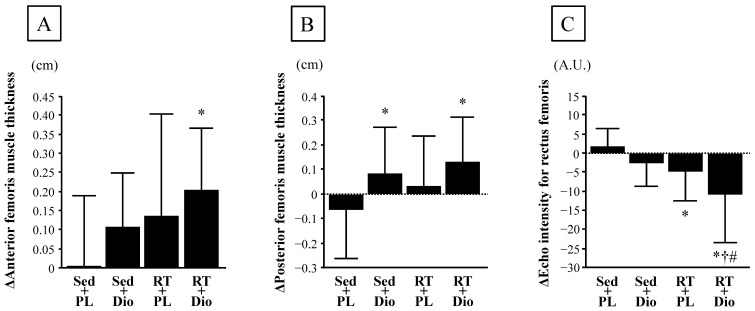
Change in anterior femoris muscle thickness (**A**), posterior femoris muscle thickness (**B**), and echo intensity for rectus femoris (**C**) before and after 12-week intervention period in sedentary-control and placebo intake (Sed and PL), sedentary-control and *Dioscorea* intake (Sed and Dio), resistance-training and Placebo intake (RT and PL), and resistance-training and *Dioscorea* intake (RT and Dio) groups. A.U., arbitrary units. Data are expressed as means ± SD. * *p* < 0.05 vs. Sed and PL group; ^†^ *p* < 0.05 vs. Sed and Dio; ^#^ *p* < 0.05 vs. RT and PL.

No significant differences were observed in anterior and posterior femoris muscle thickness, echo intensity for the rectus femoris, normal walking speed, single-leg stance test, five times sit-to-stand test, and grip strength before and after the interventions among the four groups (Table 3). Changes in anterior femoris muscle thickness from baseline to 12 weeks did not differ among the Sed and PL, Sed and Dio, and RT and PL groups, whereas the change in anterior femoris muscle thickness in the RT and Dio group was significantly greater than that in the Sed and PL group (Figure 2A and Table 3).

Furthermore, changes in the posterior femoris muscle thickness from baseline to 12 weeks in the Sed and Dio, and RT and Dio groups were significantly higher than those in the Sed and PL group; however, these changes did not differ between the Sed and PL, and RT and PL groups (Figure 2B and Table 3). Additionally, changes in the echo intensity of the rectus femoris from baseline to 12 weeks in the RT and PL, and RT and Dio groups were significantly lower than those in the Sed and PL group, and the changes in the echo intensity of the rectus femoris in the RT and Dio group were significantly lower than those in the Sed and Dio, and RT and PL groups (Figure 2C and Table 3). Changes in five times sit-to-stand test from baseline to 12 weeks did not differ among the Sed and PL, Sed and Dio, and RT and PL groups, whereas the change in the five times sit-to-stand test in the RT and Dio group was significantly lower than that in the Sed and PL group (Table 3). However, no significant differences in normal walking speed, single-leg stand test, and grip strength from baseline to 12 weeks were observed among the four groups (Table 3).

**Figure 3:**
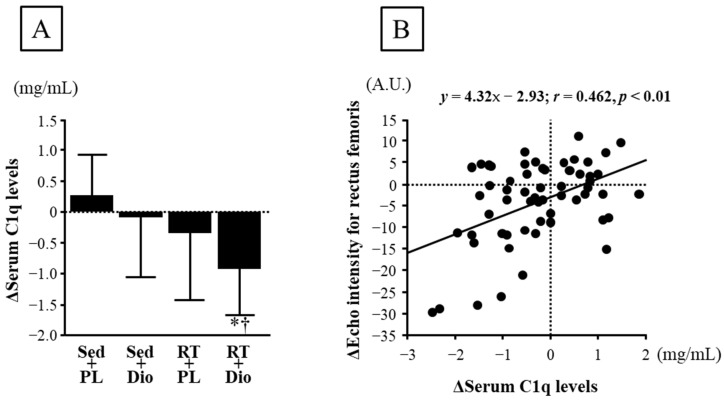
(**A**): Change in serum C1q level before and after 12-week intervention in Sed and PL, Sed and Dio, RT and PL, and RT and Dio groups. A.U., arbitrary units. Data are expressed as means ± SD. * *p* < 0.05 vs. Sed and PL group, † *p* < 0.05 vs. Sed and Dio. (**B**): Association between change in serum C1q level and echo intensity for rectus femoris before and after 12-week interventions (*n* = 60). r = Pearson’s correlation coefficient.

Changes in the serum C1q levels from baseline to 12 weeks did not differ among the Sed and PL, Sed and Dio, and RT and PL groups, whereas changes in the serum C1q levels in the RT and Dio group were significantly lower than those in the Sed and PL, and Sed and Dio groups (Figure 3A). Furthermore, the change in serum C1q levels from baseline to 12 weeks significantly correlated with the change in echo intensity of the rectus femoris (Figure 3B).

### 3.3. Cardiometabolic Parameters

No significant differences in SBP, DBP, HR, Total-Cho, HDL, TG, or HbA1c before and after the interventions were observed among the four groups (Table 4). Changes in DBP from baseline to 12 weeks in the Sed and Dio, RT and PL, and RT and Dio groups were significantly lower than those in the Sed and PL group (Table 4). Furthermore, the changes in HbA1c from baseline to 12 weeks did not differ between the Sed and PL, and RT and PL groups, whereas the change in HbA1c in the Sed and Dio, and RT and Dio groups was significantly lower than that in the Sed and PL group (Table 4).

## 4. Discussion

Our main findings demonstrated that in healthy middle-aged and older adults, 12-week *Dioscorea esculenta* intake alone increased posterior femoris muscle thickness, while 12-week low-intensity resistance exercise alone improved muscle echo intensity but did not significantly affect other measurements. Interestingly, 12-week *Dioscorea esculenta* intake combined with low-intensity resistance exercise induced greater improvements in femoris muscle thickness, echo intensity for the rectus femoris (index of muscle quality), and five times sit-to-stand test (index of muscle function) compared to being sedentary with placebo intake, while the echo intensity further improved compared to the effects observed with *Dioscorea esculenta* intake or low-intensity resistance exercise alone. Therefore, chronic *Dioscorea esculenta* intake combined with low-intensity resistance exercise may more effectively improve muscle quantity and quality indices in healthy middle-aged and older adults. 

In the present study, 12-week *Dioscorea esculenta* intake combined with low-intensity resistance exercise in healthy middle-aged and older adults resulted in greater femoris muscle thickness and a faster test of five times sit-to-stand (an index of muscle function) than that associated with sedentary participants with placebo intake, though low-intensity resistance exercise alone did not clearly affect them. Enhanced muscle protein synthesis via the mTOR/p70S6K signaling pathway activated through chronic repeated acute resistance exercise results in muscle hypertrophy [34]. Our recent study in rats showed that acute resistance exercise-induced activation of the Akt/mTOR/p70S6K signaling pathway with an increase in muscle DHT production was attenuated through the pre-administration of a DHT synthase inhibitor [35]. Furthermore, continuous administration of DHT synthase inhibitors during regular resistance exercise suppresses muscle hypertrophy [21]. Additionally, habitual resistance exercise in the elderly increases the expression of steroidogenic enzymes in skeletal muscle and muscle DHT levels, and muscle DHT is correlated with muscle cross-sectional area [20]. In our previous study, the chronic intake of diosgenin, which has a chemical structure similar to that of DHEA, increased muscle DHT production [23]. Moreover, chronic diosgenin intake partially increased muscle mass, which was suppressed through the continuous administration of DHT synthase inhibitor [23]. Therefore, *Dioscorea esculenta* intake combined with low-intensity resistance exercise in healthy middle-aged and older adults may accelerate the increase in muscle DHT secretion, leading to muscle hypertrophy and functional improvement. In fact, in our recent study on athletes, chronic intake of 2000 mg of *Dioscorea esculenta* with resistance training partially increased muscle mass and strength compared with resistance training alone [24]. Thus, chronic *Dioscorea esculenta* supplementation plays a crucial role in influencing the effects of resistance training on muscle mass and function.

Aging accelerates muscle fibrosis, which results in decreased muscle quality [7,8]. This mechanism involves the activation of muscle Wnt signaling via an age-related increase in systemic C1q levels [36]. Recently, we demonstrated that chronic resistance exercise in aged mice reduced age-related enhancement of muscle C1q production and, thus, may contribute to reduced aging-induced muscle fibrosis [9]. Therefore, C1q is a potential biomarker of muscle fibrosis. In this study, 12-week *Dioscorea esculenta* intake combined with low-intensity resistance exercise further improved femoris echo intensity (an index of muscle quality) and circulating C1q levels compared to being sedentary with placebo or *Dioscorea esculenta* intake in healthy middle-aged and older adults. Furthermore, changes in circulating C1q levels were positively associated with changes in muscle echo intensity. Therefore, *Dioscorea esculenta* intake combined with low-intensity resistance exercise in healthy middle-aged and older adults may further reduce C1q production, leading to improved muscle quality. Additionally, in this study, we used muscle echo intensity as an index of muscle quality to evaluate muscle adipose and fibrous connective tissues. Therefore, it is considered that the reduction in both muscle adipose and fibrous connective tissues is involved in the reduction in muscle echo intensity through a combination of *Dioscorea esculenta* intake and low-intensity resistance exercise. Our previous animal study indicated that *Dioscorea esculenta* supplementation enhanced the enzymatic activity of skeletal muscle oxidative phosphorylation [23]. Therefore, a reduction in muscle fat infiltration via increased muscle fat utilization induced using diosgenin may be involved in the improvement of muscle quality.

This study revealed that, among the cardiometabolic risk factors, HbA1c decreased in the sedentary and low-intensity resistance-trained groups with *Dioscorea esculenta* intake. Our previous study showed that chronic intake of *Dioscorea esculenta* improves glycemic control through promoting glucose uptake and utilization mediated via the activated phosphorylation of the Akt/PKC/GLUT4 signaling pathway in skeletal muscle [23]. In addition, the continuous administration of DHT synthase inhibitor suppressed these effects [23]. Chronic DHEA administration promotes glucose uptake and utilization through accelerating GLUT4 translocation in skeletal muscles, increasing muscle DHT secretion [37]. Thus, the supply of diosgenin abundantly present in *Dioscorea esculenta* may promote glucose metabolism and the utilization of the skeletal muscle through assisting the secretion of sex steroid hormones in the skeletal muscle, resulting in a decrease in HbA1c.

In this study, 12-week resistance training with and without *Dioscorea esculenta* intake and *Dioscorea esculenta* intake alone decreased the DBP compared to the effects of placebo in sedentary middle-aged and older adults. DHEA can metabolize estrogen from testosterone [38]. As estrogen has a vasodilatory effect [39], increased estrogen production due to *Dioscorea esculenta* intake may be involved in lowering blood pressure. Additionally, in a meta-analysis, habitual resistance exercise was shown to reduce blood pressure [40,41]; however, the effect of this exercise remains unclear. Thus, habitual low-intensity resistance exercise and *Dioscorea esculenta* intake may reduce DBP.

In middle-aged and older adults, performing high-intensity exercise is difficult and places a heavy burden not only on the joints and muscles, but also on the circulatory system. In contrast, low-intensity exercise is easier to continue; however, its effects may be more limited. In this study, *Dioscorea esculenta* intake combined with low-intensity resistance exercise further improved muscle thickness, echo intensity, and function in healthy middle-aged and older adults, compared to each intervention alone. Therefore, chronic intake of *Dioscorea esculenta* could be used as a new therapeutic strategy for middle-aged and older adults to enhance the effects of exercise on muscle quantity and quality.

This study had five limitations. Firstly, we believe that the effects of resistance training with *Dioscorea esculenta* intake may involve the action of diosgenin; however, other components contained in *Dioscorea esculenta* may also be related to these effects. Secondly, this study did not confirm whether diosgenin-mediated steroidogenesis, such as DHEA, testosterone, and DHT, is promoted in skeletal muscle. Thirdly, although low-intensity resistance training with *Dioscorea esculenta* was performed in this study, it is necessary to verify the effects of other exercise modes, particularly aerobic training. Fourthly, though this study showed that circulating C1q levels were associated with the effect mechanism on muscle quality in low-intensity resistance training with *Dioscorea esculenta* intake, the molecular mechanism was not elucidated. Further studies are required to examine the effects on muscle quality. Fifthly, we investigated a small number of middle-aged and older adults; however, further studies targeting other age groups and patients with disease risk are necessary.

## 5. Conclusions

In this study, we revealed that 12-week *Dioscorea esculenta* intake combined with low-intensity resistance exercise improved muscle thickness, function, and, in particular, echo intensity in healthy middle-aged and older adults, compared to the effects observed with *Dioscorea esculenta* intake or low-intensity resistance exercise alone. These findings suggest that the chronic intake of *Dioscorea esculenta* additively enhances the effects of habitual resistance exercise on muscle quantity and quality. Furthermore, 12-week *Dioscorea esculenta* intake alone improved HbA1c and DBP in healthy middle-aged and older adults. These findings suggest that chronic intake of *Dioscorea esculenta* improves some cardiometabolic parameters.

## Figures and Tables

**Table 1:** Comparison of subject characteristics before and after interventions.

	Sed+PL		Sed+Dio		RT+PL		RT+Dio		Two-Way ANOVA	ΔSed+PL	ΔSed+Dio	ΔRT+PL	ΔRT+Dio	One-Way ANOVA
	Before	After	Before	After	Before	After	Before	After						
Age, year	53 ± 5	53 ± 5	55 ± 7	55 ± 7	52 ± 5	53 ± 6	53 ± 5	53 ± 5	1.000					
Height, cm	161 ± 8	161 ± 7	159 ± 9	159 ± 9	160 ± 9	160 ± 9	161 ± 5	161 ± 5	1.000					
Body weight, kg	59 ± 9	59 ± 9	61 ± 12	61 ± 11	61 ± 14	60 ± 14	64 ± 10	63 ± 9	1.000	0.2 ± 1.2	0.3 ± 1.0	−0.4 ± 0.8	−0.1 ± 1.0	0.289
BMI, kg/cm^2^	23 ± 3	23 ± 3	24 ± 4	24 ± 4	23 ± 4	23 ± 4	24 ± 4	25 ± 4	0.998	0.1 ± 0.4	0.3 ± 0.4	−0.1 ± 0.4	0.1 ± 0.4	0.104
Body fat, %	28 ± 6	28 ± 6	31 ± 8	32 ± 8	30 ± 7	31 ± 7	30 ± 10	31 ± 9	0.991	0.3 ± 1.7	0.6 ± 1.6	0.3 ± 1.7	0.3 ± 1.1	0.949
PA, kcal/day	789 ± 392	824 ± 415	817 ± 374	710 ± 267	731 ± 451	716 ± 362	656 ± 312	688 ± 338	0.865	35 ± 221	−107 ± 369	−15 ± 260	32 ± 191	0.439
EI, kcal/day	1496 ± 480	1515 ± 500	1446 ± 476	1622 ± 525	1677 ± 626	1604 ± 627	1508 ± 505	1666 ± 486	0.773	19 ± 484	176 ± 256	−73 ± 299	159 ± 276	0.155

Data are means and SD. Sed+PL: sedentary-control and placebo intake group, Sed+Dio: sedentary-control and *Dioscorea* intake group, RT+PL: resistance-training and placebo intake group, RT+Dio: resistance-training and *Dioscorea* intake group, BMI: body mass index, PA: daily physical activity, EI: daily total energy intake.

**Table 2:** Nutritional status before and after interventions.

	Sed+PL		Sed+Dio		RT+PL		RT+Dio		Two-Way ANOVA	ΔSed+PL	ΔSed+Dio	ΔRT+PL	ΔRT+Dio	One-Way ANOVA
	Before	After	Before	After	Before	After	Before	After						
Protein (g/day)	60 ± 26	57 ± 23	62 ± 26	62 ± 24	60 ± 18	60 ± 22	65 ± 23	62 ± 19	0.992	−2.6 ± 13.7	−0.1 ± 14.3	−0.3 ± 12.7	−3.1 ± 17.9	0.924
Fat (g/day)	48 ± 20	46 ± 16	57 ± 19	57 ± 19	53 ± 18	49 ± 17	54 ± 17	51 ± 15	0.966	−2.1 ± 12.3	0.6 ± 11.9	−3.8 ± 14.3	−3.3 ± 12.8	0.794
Carbohydrate (g/day)	190 ± 66	193 ± 59	200 ± 84	192 ± 95	204 ± 111	183 ± 82	201 ± 59	209 ± 69	0.913	2 ± 28	−8 ± 58	−20 ± 62	8 ± 46	0.448
Sodium (g/day)	4 ± 1	3 ± 1	4 ± 1	3 ± 1	3 ± 1	3 ± 1	4 ± 1	4 ± 1	0.995	−0.1 ± 0.8	−0.1 ± 0.8	−0.2 ± 0.7	−0.1 ± 1.0	0.950
Potassium (mg/day)	2120 ± 947	2185 ± 855	2102 ± 808	1976 ± 740	2280 ± 815	2243 ± 939	2263 ± 949	2084 ± 664	0.948	65 ± 463	−126 ± 413	−37 ± 500	−180 ± 627	0.575
Calcium (mg/day)	434 ± 177	385 ± 134	465 ± 230	423 ± 196	474 ± 175	499 ± 189	472 ± 234	416 ± 152	0.991	−49 ± 131	−42 ± 162	−25 ± 95	−56 ± 145	0.935
Magnesium (mg/day)	214 ± 91	216 ± 83	224 ± 89	210 ± 83	230 ± 68	221 ± 86	221 ± 86	211 ± 69	0.986	2 ± 38	−14 ± 43	−9 ± 45	−9 ± 61	0.832
Iron (mg/day)	7 ± 4	7 ± 3	7 ± 3	7 ± 3	7 ± 3	7 ± 3	7 ± 3	7 ± 2	0.997	−0.4 ± 2.1	−0.3 ± 1.3	−0.1 ± 1.5	−0.4 ± 2.3	0.971
Phosphorus (mg/day)	894 ± 383	841 ± 313	924 ± 390	901 ± 366	915 ± 263	886 ± 330	966 ± 367	912 ± 277	0.997	53 ± 219	−23 ± 218	−29 ± 160	−53 ± 251	0.970
Vitamin D (μg/day)	12 ± 10	9 ± 5	10 ± 8	9 ± 7	9 ± 6	10 ± 9	10 ± 8	11 ± 5	0.592	−3.8 ± 8.2	−0.6 ± 4.7	1.1 ± 6.0	0.2 ± 5.9	0.179
Vitamin E (mg/day)	7 ± 3	7 ± 2	7 ± 2	7 ± 3	7 ± 3	7 ± 4	7 ± 3	7 ± 3	0.998	−0.1 ± 1.8	0.2 ± 1.4	−0.1 ± 1.7	−0.1 ± 2.1	0.971
Vitamin C (mg/day)	86 ± 47	101 ± 52	73 ± 29	74 ± 34	88 ± 58	99 ± 56	85 ± 50	91 ± 49	0.948	15 ± 34	1 ± 24	11 ± 41	6 ± 34	0.701
Folic acids (μg/day)	307 ± 192	291 ± 110	286 ± 110	266 ± 112	305 ± 176	310 ± 166	308 ± 176	284 ± 103	0.982	−16 ± 153	−19 ± 61	5 ± 87	−23 ± 112	0.894
Zinc (mg/day)	7 ± 3	7 ± 3	7 ± 2	7 ± 3	7 ± 2	7 ± 2	8 ± 2	7 ± 2	0.994	−0.2 ± 1.6	0.1 ± 1.7	−0.3 ± 1.4	−0.3 ± 2.0	0.961
Cholesterol (mg/day)	344 ± 174	292 ± 116	411 ± 229	423 ± 246	360 ± 143	348 ± 159	442 ± 201	401 ± 128	0.899	−53 ± 131	12 ± 95	−13 ± 104	−41 ± 147	0.460
Dietary fiber (g/day)	10 ± 4	11 ± 4	10 ± 4	9 ± 4	11 ± 5	11 ± 6	10 ± 5	10 ± 4	0.932	0.7 ± 1.7	−0.9 ± 1.9	−0.1 ± 2.7	−0.1 ± 3.6	0.435

Data are means and SD. Sed+PL: sedentary-control and placebo intake group, Sed+Dio: sedentary-control and *Dioscorea* intake group, RT+PL: resistance-training and placebo intake group, RT+Dio: resistance-training and *Dioscorea* intake group.

**Table 3:** Comparison of muscle quantity and quality before and after interventions.

	Sed+PL		Sed+Dio		RT+PL		RT+Dio		Two-Way ANOVA	ΔSed+PL	ΔSed+Dio	ΔRT+PL	ΔRT+Dio	One-Way ANOVA
	Before	After	Before	After	Before	After	Before	After						
Anterior femoris muscle thickness, cm	4.3 ± 0.6	4.3 ± 0.6	4.3 ± 0.7	4.4 ± 0.7	3.9 ± 0.7	4.1 ± 0.7	4.3 ± 0.6	4.5 ± 0.6	0.948	0.01 ± 0.19	0.11 ± 0.14	0.14 ± 0.27	0.21 ± 0.16 *	0.046
Posterior femoris muscle thickness, cm	4.9 ± 0.8	4.9 ± 0.8	5.3 ± 0.9	5.4 ± 0.9	5.1 ± 0.7	5.2 ± 0.6	5.1 ± 0.5	5.3 ± 0.6	0.959	−0.07 ± 0.20	0.08 ± 0.19 *	0.03 ± 0.20	0.13 ± 0.18 *	0.044
Echo intensity for rectus femoris, A.U.	47.1 ± 14.1	49.0 ± 14.6	55.4 ± 22.3	52.8 ± 22.6	54.8 ± 16.8	50.2 ± 12.7	50.0 ± 23.0	39.3 ± 16.8	0.604	1.9 ± 4.7	−2.6 ± 6.2	−4.6 ± 7.9 *	−10.8 ± 12.7 *†#	0.001
Normal walking speed, m/s	1.5 ± 0.2	1.5 ± 0.2	1.5 ± 0.1	1.5 ± 0.1	1.5 ± 0.2	1.6 ± 0.2	1.5 ± 0.1	1.5 ± 0.2	0.723	0.05 ± 0.16	0.01 ± 0.16	0.10 ± 0.14	0.05 ± 0.13	0.379
Single leg stand test, min	1.6 ± 0.6	1.8 ± 0.4	1.6 ± 0.7	1.6 ± 0.6	1.4 ± 0.7	1.8 ± 0.4	1.6 ± 0.6	1.8 ± 0.4	0.691	7.5 ± 28.4	2.0 ± 21.3	22.3 ± 30.5	12.4 ± 21.5	0.180
Five times sit to stand test, sec	7.8 ± 1.3	7.2 ± 1.1	7.8 ± 0.8	6.9 ± 1.1	8.6 ± 1.0	7.5 ± 1.0	8.5 ± 1.2	7.0 ± 1.2	0.426	−0.6 ± 0.9	−0.9 ± 0.8	−1.1 ± 0.9	−1.5 ± 1.1 *	0.049
Grip strength, kg	26.3 ± 7.0	26.5 ± 5.9	25.8 ± 9.0	26.0 ± 9.2	26.3 ± 8.9	26.9 ± 8.0	27.7 ± 7.4	29.2 ± 7.1	0.987	0.2 ± 1.7	0.2 ± 2.0	0.6 ± 2.7	1.5 ± 2.4	0.349

Data are means and SD. Sed+PL: sedentary-control and placebo intake group, Sed+Dio: sedentary-control and *Dioscorea* intake group, RT+PL: resistance-training and placebo intake group, RT+Dio: resistance-training and *Dioscorea* intake group, A.U.: arbitrary unit. * *p* < 0.05 vs. ΔSed+PL, † *p* < 0.05 vs. ΔSed+Dio, # *p* < 0.05 vs. ΔRT+PL.

**Table 4:** Comparison of cardiometabolic parameters before and after interventions.

	Sed+PL		Sed+Dio		RT+PL		RT+Dio		Two-Way ANOVA	ΔSed+PL	ΔSed+Dio	ΔRT+PL	ΔRT+Dio	One-Way ANOVA
	Before	After	Before	After	Before	After	Before	After						
SBP, mmHg	123 ± 19	121 ± 21	121 ± 16	115 ± 21	123 ± 12	117 ± 22	127 ± 23	120 ± 19	0.968	0.2 ± 10.6	−4.1 ± 8.9	−4.5 ± 14.8	−7.1 ± 11.6	0.394
DBP, mmHg	74 ± 10	75 ± 12	74 ± 15	68 ± 12	76 ± 7	68 ± 10	79 ± 16	74 ± 14	0.604	1.7 ± 9.3	−4.5 ± 6.3 *	−7.0 ± 6.7*	−4.9 ± 9.3 *	0.028
HR, bpm	76 ± 11	73 ± 6	74 ± 12	70 ± 13	74 ± 10	74 ± 9	76 ± 7	79 ± 7	0.535	−2.9 ± 10.5	−3.8 ± 10.6	−0.9 ± 6.4	2.7 ± 7.0	0.194
Total-Cho, mg/mL	214 ± 43	205 ± 37	231 ± 45	235 ± 39	233 ± 30	223 ± 38	211 ± 24	202 ± 24	0.864	−9.4 ± 26.2	3.8 ± 21.4	−9.4 ± 19.3	−8.6 ± 17.9	0.261
HDL, mg/mL	71 ± 15	69 ± 12	69 ± 20	71 ± 20	76 ± 16	73 ± 17	67 ± 21	64 ± 21	0.972	−2.1 ± 7.3	1.1 ± 6.4	−2.8 ± 5.0	−2.7 ± 2.8	0.206
TG, mg/mL	92 ± 46	88 ± 46	93 ± 46	94 ± 36	99 ± 55	90 ± 50	101 ± 48	102 ± 44	0.972	−3.9 ± 19.1	0.8 ± 30.2	−8.7 ± 14.8	1.5 ± 12.1	0.492
HbA1c, %	5.3 ± 0.2	5.4 ± 0.3	5.3 ± 0.2	5.3 ± 0.2	5.3 ± 0.3	5.3 ± 0.3	5.3 ± 0.3	5.3 ± 0.3	0.648	0.09 ± 0.19	−0.03 ± 0.14 *	−0.01 ± 0.14	−0.08 ± 0.14 *	0.037

Data are means and SD. Sed+PL: sedentary-control and placebo intake group, Sed+Dio: sedentary-control and *Dioscorea* intake group, RT+PL: resistance-training and placebo intake group, RT+Dio: resistance-training and *Dioscorea* intake group, SBP: systolic blood pressure, DBP: diastolic blood pressure, HR: heart rate, Total-Cho: serum total cholesterol concentration, HDL: serum high-density lipoprotein cholesterol concertation, TG: serum triglyceride concentration, HbA1c: glycated hemoglobin. * *p* < 0.05 vs. ΔSed+PL.

## Data Availability

The data presented in this study are available upon request from the corresponding author.
